# Locally Different Endothelial Nitric Oxide Synthase Protein Levels in Ascending Aortic Aneurysms of Bicuspid and Tricuspid Aortic Valve

**DOI:** 10.1155/2012/165957

**Published:** 2012-06-18

**Authors:** Salah A. Mohamed, Arlo Radtke, Roza Saraei, Joern Bullerdiek, Hajar Sorani, Rolf Nimzyk, Antje Karluss, Hans H. Sievers, Gazanfer Belge

**Affiliations:** ^1^Department of Cardio and Thoracic Vascular Surgery, University Clinic of Schleswig-Holstein (UKSH), Campus Luebeck, Ratzeburger Allee 160, 23538 Luebeck, Germany; ^2^Center for Human Genetics, University of Bremen, Leobener Strasse ZHG, 28359 Bremen, Germany

## Abstract

*Aims*. Dysregulated expression of the endothelial nitric oxide synthase (eNOS) is observed in aortic aneurysms associated with bicuspid aortic valve (BAV). We determined eNOS protein levels in various areas in ascending aortic aneurysms. *Methods and Results*. Aneurysmal specimens were collected from 19 patients, 14 with BAV and 5 with tricuspid aortic valve (TAV). ENOS protein levels were measured in the outer curve (convexity), the opposite side (concavity), the distal and above the sinotubular junction (proximal) aneurysm. Cultured aortic cells were treated with NO synthesis inhibitor L-NAME and the amounts of 35 apoptosis-related proteins were determined. In patients with BAV, eNOS levels were significantly lower in the proximal aorta than in the concavity and distal aorta. ENOS protein levels were also lower in the convexity than in the concavity. While the convexity and distal aorta showed similar eNOS protein levels in BAV and TAV patients, levels were higher in TAV proximal aorta. Inhibition of NO synthesis in aneurysmal aortic cells by L-NAME led to a cytosolic increase in the levels of mitochondrial serine protease HTRA2/Omi. *Conclusion*. ENOS protein levels were varied at different areas of the aneurysmal aorta. The dysregulation of nitric oxide can lead to an increase in proapoptotic HTRA2/Omi.

## 1. Introduction

Bicuspid aortic valve (BAV) is a common congenital cardiac defect having a prevalence of 0.9% to 2%. It is associated with stenosis, insufficiency, and ascending aortic aneurysms [1]. The formation of ascending aortic aneurysms in BAV patients seems to be linked to apoptosis of medial vascular smooth muscle cells (VSMCs). Apoptosis was found to be more frequent in the medial tissue of dilated aortas and cultured VSMCs derived from aneurysmatic aortas. Additionally cultured VSMCs derived from BAV dilated aortas showed higher apoptosis rates than VSMCs from control patients [2, 3].

The endothelial nitric oxide synthase (eNOS) is associated with the development of BAV, and eNOS-produced nitric oxide (NO) is also believed to play a role in aneurysm formation. In 2000 Lee and colleagues showed that eNOS-deficient mice were predisposed to develop a BAV, whereas Kuhlencordt et al. found a higher incidence of aortic aneurysm in eNOS/Apolipoprotein E double-knockout mice [4, 5]. A study by Aicher et al. presented a significant decrease in eNOS protein amount in BAV aortic tissue compared to TAV aortic tissue [6]. Expression and activity of eNOS in aortic endothelial cells are controlled by hemodynamic wall shear stress [7, 8]. Recent publications have indicated that aortic wall shear stress differs locally between BAV and control patients when examined via magnetic resonance imaging (MRI) [9, 10]. Furthermore we provided evidence that VSMCs show a different apoptotic behavior in the concave versus the convex side of the dilated aorta. Inhibition of caspase-3 was shown to protect cultured cells derived from the concavity of the aorta more significantly than those from the convexity [11]. Taken together, these results suggest a locally different behavior of medial cells in BAV aortic tissue than in TAV aortic tissue [12]. This is particularly interesting because it is known that eNOS-derived NO can inhibit caspases via S-nitrosylation [13], which might explain the observed differences in apoptosis between the different aortic areas. Therefore, we determined eNOS protein levels at different aortic areas of aneurysmal BAV and TAV patients. We also determined the effects of NO-synthesis inhibition on apoptosis of primary human aortic endothelial cells (HAECs) and human aortic VSMCs taken from tissue of a BAV patient.

## 2. Methods

### 2.1. Sample Collection

The investigation conforms with the principles outlined in the Declaration of Helsinki for use of human tissue. The study protocol was approved by the institutional ethics committee, and written informed consent was obtained from all patients. Specimens of aortic wall of 14 BAV and 5 TAV aneurysmal patients were collected during surgery (patient characteristics are shown in Table 1). The tissue was taken from 4 distinct areas of the aorta, the concave side of the aorta (inner curves), the convex side of the aorta (outer curves, the right anterolateral aspect), the distal aneurysm (under the aortic arch), and the proximal aneurysm (above the sinotubular junction), and immediately placed in liquid nitrogen. Cell lysate was prepared using the Bio-Plex Cell Lysis Kit according to the manufacturer's instructions (BIO-Rad, Hamburg, Germany). Whole protein concentration in the cell lysate was measured using the BCA protein assay (Thermo Scientific, Rockford, USA). 

### 2.2. Western Blot Analysis

The wells of NuPAGE 10% Bis-Tris gels (Invitrogen, Darmstadt, Germany) were loaded with 20 *μ*g of cell lysate, and a denaturising SDS-PAGE was run using the Xcell SureLock Mini-cell (Invitrogen, Darmstadt, Germany) and appropriate buffers. The separated proteins were transferred to a nitrocellulose membrane, which was blocked with TBS-T containing 5% (v/w) BSA at 4°C overnight. The membrane was washed thrice in TBS-T and incubated with primary antibody diluted in TBS-T containing 5% BSA (v/w) for 1 h. Then, the washing step was repeated, and the membrane was treated with secondary antibody in TBS-T containing 5% BSA (v/w) for 1 h. NOS3 rabbit polyclonal antibody (sc-654) was used together with bovine anti-rabbit IgG-AP (sc-2372) secondary antibody (Santa Cruz Biotec, Heidelberg, Germany) to detect eNOS protein. The membrane was washed thrice with TBS-T and thrice with TBS before protein bands were visualized by incubation of the membrane in staining solution made of 300 *μ*L NBT/BCIP stock solution (Roche, Mannheim, Germany) and 15 mL substrate buffer (0.1 M Tris-HCl, pH 9.5, 0.1 M NaCl, 0.05 M MgCl_2_). 

Band intensity was analyzed using the imageJ software. To compare band intensity between different blots, a calibrator sample was run on every blot. 

Both beta-actin (Novus Biologicals, Heford, Germany) and GAPDH (Santa Cruz Biotec, Heidelberg, Germany) were tested as loading control. Both controls displayed a high fluctuation in band intensity. Therefore the initial loading amount was used to normalize eNOS band intensity. 

### 2.3. Cell Culture

Vascular smooth muscle cells (VSMCs) were taken from the *tunica media* of a BAV patient. For cell culture this sample was minced and treated with 0.26% collagenase (250 U/mL, Serva, Heidelberg, Germany) at 37°C for about 3-4 h. After centrifugation, the pellet was resuspended in culture medium (TC199 with Earle's salts supplemented with 20% fetal bovine serum, 200 U/mL penicillin, 200 *μ*g/mL streptomycin) and incubated at 37°C in 5% CO_2_ air. After attachment of the tissue pieces 5 mL fresh medium were added. The monolayer culture was passaged by standard trypsine dispersion and resuspended in TC199 culture medium. 

Primary human aortic endothelial cells (HAECs) were purchased from Lonza and cultured in endothelial cell growth medium-2 (Lonza, Cologne, Germany).

### 2.4. Transfection and Characterization of Human Aortic VSMCs 

Human aortic VSMCs obtained from a first passage monolayer culture were plated in 25 cm² flasks and allowed to attach and grow for 48 h until the culture reached approximately 80% confluency. The subconfluent muscle cell culture was then transfected with 5 *μ*g of pSV40-dN-plasmid DNA as described previously [14, 15] using Effectene kit (Qiagen; Hilden, Germany). Culture supernatant containing transfecting solution was removed after 1 h. Cells were washed with PBS and cultured in TC199 medium containing 20% FCS and 2% (v/v) antibiotic solution. When cultures showed focus formation, foci were isolated and subcultured in TC199 culture medium containing 20% FCS and 2% (v/v) antibiotic solution. The population doubling time was measured as described previously [14]. 

For cell characterization, cytosolic proteins were extracted and analyzed in western blot. In short cells were washed with 1x PBS (4°C), and 500 *μ*L of cell lysis buffer (150 mM NaCl, 50 mM TrisHCl, 0.5% deoxycholic acid, 1% NP-40) containing 1x complete mini protease inhibitor (Roche, Mannheim, Germany) were added. A cell scraper was used to remove the VSMCs from the cell culture flask, and the suspension was incubated on a shaker on ice for 35 min. Subsequently the cell lysate was centrifuged at 14,200 ×g and 4°C for 20 min to collect the cytosolic protein fraction in the supernatant. The whole protein amount was determined using the BCA protein assay (Thermo Scientific, Rockford, USA). Western blot analysis was performed as described above. A smooth muscle alpha-actin primary antibody (Mab 1420; R&D Systems, Wiesbaden-Nordenstadt, Germany) was used as a smooth muscle cell marker, and a von Willebrand factor primary antibody (sc-53466; Santa Cruz, Heidelberg. Germany) was used as marker for endothelium. Since cells did contain smooth muscle alpha-actin but not von Willebrand factor (data not shown) they were considered as vascular smooth muscle cells (VSMCs).

### 2.5. L-NAME Treatment

VSMCs and HAECs were grown to 90% confluency and then incubated in serum-free medium for 24 h. Afterwards cells were treated with 1 mM of NO synthase inhibitor L-NAME for one hour. The cytosolic protein fraction was extracted as described above.

### 2.6. Quantification of Apoptosis-Related Proteins

For quantification of 35 apoptosis-related proteins after L-NAME treatment, 200 *μ*g of cytosolic protein were used in conjunction with the proteome profiler array (R&D Systems, Wiesbaden-Nordenstadt, Germany) according to the manufacturer's instructions. In short, the arrays were blocked for 1 h and then incubated with 200 *μ*g protein lysate at 4°C over night. Afterwards the arrays were incubated with biotinylated detection antibody cocktail (1 : 1000, R&D Systems, Wiesbaden-Nordenstadt, Germany) for one hour, and antibiotin alkaline phosphatase conjugated secondary antibody (1 : 10,000, Sigma-aldrich, Munich, Germany) for 30 minutes. Protein spots were visualized with solution made of 300 *μ*L NBT/BCIP stock solution (Roche, Mannheim, Germany) and 15 mL substrate buffer (0.1 M Tris-HCl, pH 9.5, 0.1 M NaCl, 0.05 M MgCl_2_). Spot intensity was quantified using the imagej software. The spots of the positive control were used as calibrator. Data were taken from three independent experiments with two replicates each. 

### 2.7. Statistical Analysis

Statistical analysis was done with SPSS and Excel/WinSTAT, and all data are displayed as absolute numbers and relative percentages or as mean ± standard deviation. Differences in eNOS protein expression were tested with two-sided Mann—Whitney U-test for independent variables (comparison between BAV and TAV areas) or with Wilcoxon signed-rank test for dependent variables (comparison between different BAV or TAV areas). Differences in apoptosis-related proteins after L-NAME treatment were analysed with repeated measurement two-way ANOVA to account for the two replicates on each apoptosis array. Relative frequencies were compared using the fisher exact test. Significant differences were assumed at *P* < 0.05.

## 3. Results

### 3.1. Determination of eNOS Protein Levels in Different Aortic Areas

Aortic tissue was taken during aneurysmal replacement from 14 patients with BAV and from 5 patients with TAV. Patients with BAV tended to be younger, having a low BMI, smaller aortic diameter with a higher percentage of aortic stenosis and combined stenosis/insufficiency but a significantly lower percentage of aortic insufficiency (21.4% versus 100%, *P* = 0.005). However, none of these characteristics differed significantly along with gender distribution and the percentage of aortic insufficiency (see Table 1). So we assumed these patient groups to be comparable. 

**Figure 1:**
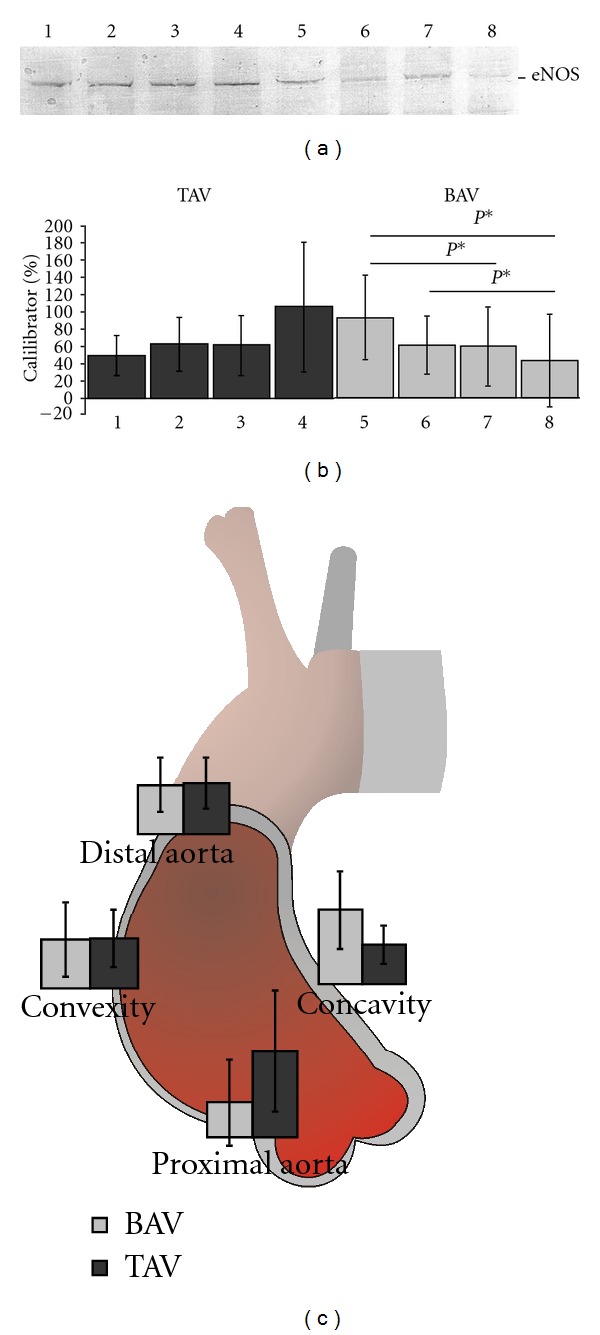
(a) Representative western blot of pooled cell lysate from 4 TAV and 4 BAV patients. 1: TAV concavity; 2: TAV distal aneurysm; 3: TAV convexity; 4: TAV proximal aneurysm; 5: BAV concavity; 6: BAV distal aneurysm; 7: BAV convexity; 8: BAV proximal aneurysm. (b) The eNOS protein level ± SD in different aortic areas was determined by densitometry. 1: TAV concavity; 2: TAV distal aneurysm; 3: TAV convexity; 4: TAV proximal aneurysm; 5: BAV concavity; 6: BAV distal aneurysm; 7: BAV convexity; 8: BAV proximal aneurysm; P*: *P* < 0.05; TAV: *n* = 5; BAV: *n* = 14. (c) eNOS protein levels in different areas of TAV and BAV aneurysmal aorta. Mean levels of eNOS protein amounts ± SD are given for each aortic area. Differences between eNOS protein levels of TAV (*n* = 5) and BAV (*n* = 14) in the proximal aneurysmal aorta are significant with *P* = 0.04.

Protein levels of eNOS were quantified in the concavity, distal aneurysm, convexity, and proximal aneurysm of these BAV and TAV patients (Figure 1). 

 In TAV patients, eNOS protein level was least in the concavity, at a medium level in the distal aorta and convex side and highest in the proximal aneurysmal aorta. Nonetheless there was no significant difference in eNOS amounts between the TAV aneurysmal aortic areas. BAV patients on the other hand featured the highest eNOS protein levels in the concavity, while distal aneurysmal aorta and convexity showed medium levels and the proximal aneurysmal aorta showed low eNOS protein level. Several of these differences proved to be significant (concavity versus convexity, 93.14 ± 48.47 versus 59.67 ± 46.03% of calibrator, *P* = 0.04; concavity versus proximal aorta, 93.14 ± 48.47 versus 43.00 ± 53.38% of calibrator, *P* = 0.02; distal versus proximal aorta, 61.2 ± 31.8 versus 43.00 ± 53.38% of calibrator, *P* = 0.03) (Figure 1(b)). 

The eNOS protein amount in the TAV concave side of the aorta was lower than that in the BAV concavity but this difference was not significant (49.57 ± 23.6 versus 93.14 ± 48.47% of calibrator, *P* = 0.07). In the distal aorta and the convex aortic side there was little difference between TAV and BAV eNOS protein amounts (distal aorta: 63.23 ± 31.99 versus 61.2 ± 31.8% of calibrator, *P* = 0.96; convexity: 61.63 ± 35.38 versus 59.67 ± 46.03% of calibrator, *P* = 0.75). However, in the proximal aorta, eNOS levels were significantly higher in TAV patients than BAV patients (105.79 ± 75.11 versus 43.00 ± 53.38% of calibrator, *P* = 0.04) (Figure 1(c)). 

### 3.2. Quantification of Apoptosis-Related Proteins after Treatment of VSMCs and HAECs with 1 mM L-NAME

**Figure 2:**
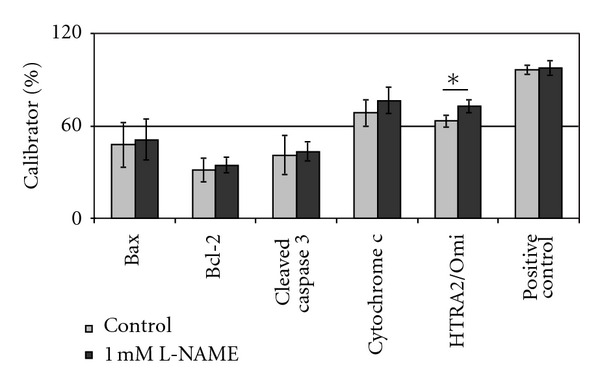
Change in concentration of apoptosis-related proteins after treatment with L-NAME. Bars display the change in concentration of Bax, Bcl-2, cleaved caspase 3, cytochrome c, HTRA2/Omi, and the positive control after treatment of VSMCs with 1 mM L-NAME for 1 h. *: *P* = 0.04.

To determine the effect a dysregulation of eNOS would have on aortic cell apoptosis, we treated VSMCs originating from aortic tissue and primary HAECs with 1 mM of the specific NO-synthase inhibitor L-NAME for one hour. Afterwards 35 apoptosis-related proteins were quantified using a proteome profiler array for human apoptosis. In VSMCs most apoptotic proteins increased in abundance, although only HTRA2/Omi levels were significantly elevated after L-NAME treatment (*P* = 0.04, Figure 2). 

Treatment with L-NAME resulted in little difference in Bax, Bcl-2, and cleaved caspase-3 concentration. The cytosolic amount of cytochrome c was slightly elevated (68.46 ± 8.61 versus 76.39 ± 8.48% of calibrator) although this difference was not significant (*P* = 0.336). Cytosolic protein levels of HTRA2/Omi on the other hand were significantly increased after inhibition of NO synthesis (63.1 ± 3.8 versus 72.6 ± 4.2% of calibrator, *P* = 0.04). For changes in other apoptosis-related proteins see Tables 2 and 3. HTRA2/Omi is a mitochondrial serine protease, which is released into the cytosol by proapoptotic members of the Bcl-2 family [15]. Here it contributes to apoptosis by cleavage of members of the inhibitor of apoptosis (IAP) family, which otherwise function as direct inhibitors of caspase-3, -7, and -9 [16, 17]. Therefore these results indicate some form of early apoptosis in VSMCs after L-NAME treatment. 

**Figure 3:**
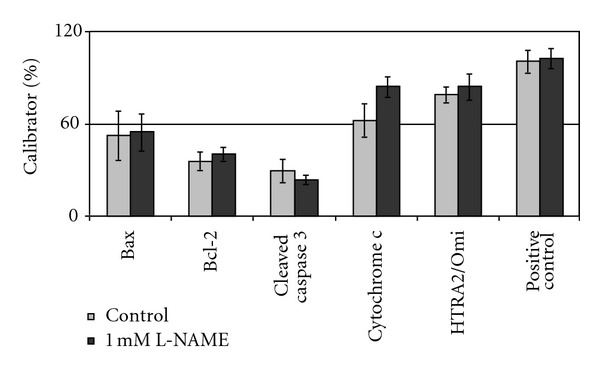
Change in concentration of apoptosis-related proteins after treatment with L-NAME. Bars display the change in concentration of Bax, Bcl-2, cleaved caspase 3, cytochrome c, HTRA2/Omi, and the positive control after treatment of HAECs with 1 mM L-NAME for 1 h.

In HAECs no significant changes in apoptosis-related proteins could be observed after L-NAME treatment (Figure 3, see also Tables 2 and 3).

Bax, Bcl-2, cleaved caspase-3, and HTRA2/Omi did not display any changes in cytosolic protein levels. Cytosolic appearance of cytochrome c increased after L-NAME treatment (62.37 ± 11.07 versus 84.29 ± 6.92% of calibrator) but this difference was not significant (*P* = 0.069).

## 4. Discussion 

The bicuspid aortic valve (BAV) is often associated with ascending aortic aneurysms. Ascending aortic aneurysms are marked by a pathologic dilatation of the aorta to the at least 1.5-fold diameter. The enzyme endothelial nitric oxide synthase (eNOS) catalyses the conversion of L-arginine to L-citrulline and nitric oxide (NO), which serves as a signaling molecule in the cardiovascular system [18, 19]. NO is known to mediate several vasoprotective properties, like inhibition of vascular smooth muscle cells (VSMCs) and proliferation and maintaining of endothelial function [20, 21]. 

Evidence for a connection between eNOS and BAV was given when Aicher et al. compared eNOS protein expression in patients with TAV and patients with BAV. They showed a downregulation of eNOS in the proximal aorta of BAV patients compared to TAV patients [6]. Our results demonstrate a varying expression of the eNOS protein in different areas of the aneurysmal aorta of BAV and TAV patients. While eNOS levels are indeed lower in the BAV proximal aorta than in its TAV counterpart, the BAV concavity features a higher eNOS expression than the TAV concavity, although this difference was not significant in our experiments (*P* = 0.07). The eNOS protein amount in the convexity and the distal aorta seems to be on equal levels in BAV and TAV. 

The upregulation of eNOS mRNA expression by fluid shear stress is well established [7]. Recently Barker et al. quantified shear stress in BAV and control patients by phase contrast MRI and thereby revealed a significant difference in aortic shear stress distribution between the groups [10]. Moreover, they found the hemodynamic forces to be significantly higher in the concavity of BAV patients than in control patients, while wall shear stress in the convexity was equal in both groups. These findings suggest an upregulation of eNOS in the BAV concavity by fluid shear stress which coincidences with our data. It is reasonable to assume that the altered valve morphology in BAV results in a different distribution of aortic wall shear stress and therefore leads to a locally different eNOS expression compared to TAV. 

Apoptosis of vascular smooth muscle cells (VSMCs) seems to account for the formation of aneurysm in BAV [2, 3, 22]. In a recent study we provided evidence that VSMCs show a different behaviour in respect to apoptosis in the concave versus the convex sites in BAV ascending aortic aneurysm [11]. We found that the inhibition of caspase 3 leads to an increased protection against apoptosis in the BAV concavity compared with the convexity. The detection of a significantly higher eNOS protein expression in the BAV concavity than in the BAV convexity appears to be consistent with these earlier results, since nitric oxide generated by eNOS is known to inhibit apoptosis by s-nitrosylation of caspases [13]. Therefore it seems that an elevated eNOS expression in the BAV concavity can contribute to an increased protection against VSMC apoptosis in this aortic area. 

To analyze the effect a dysregulation of eNOS could have on VSMC and HAEC apoptosis, we treated VSMCs which originated from aortic tissue, as well as primary HAECs with 1 mM of the NO synthase inhibitor L-NAME. Of note, L-NAME does not specifically inhibit eNOS but all NO synthases. Although eNOS is the primary source of NO in the aorta, other NO synthases are known to be active in VSMC under certain conditions [23]. Therefore the effect of L-NAME treatment cannot solely be attributed to eNOS inhibition. We are also aware that eNOS is mainly expressed in endothelial cells. However, it was shown that VSMCs do express eNOS and that the produced NO is physiologically relevant [24, 25]. Inhibition of NO synthesis in VSMC resulted in an increase in cytosolic HTRA2/Omi proteins. HTRA2/Omi is a mitochondrial serine protease, which initiates apoptosis if released into the cytosol [26]. Treatment of HAECs with L-NAME did not account for any changes in cytosolic HTRA2/Omi levels but the cytosolic amount of cytochrome c increased after NO synthase inhibition although the difference was not significant (*P* = 0.069). The increased presence of mitochondrial proteins in the cytosol after L-NAME treatment indicates an inhibitory effect of eNOS produced NO on the intrinsic apoptosis pathway. This pathway, opposed to the death receptor triggered extrinsic pathway, is activated by proapoptotic Bcl-2 proteins, which cause mitochondrial outer-membrane permeabilization and thus cause a release of mitochondrial cytochrome c into the cytosol. Here cytochrome c facilitates assembly of the apoptosome, which activates procaspase-9 and therefore initiates caspase cascades [26, 27]. We detected neither in VSMCs nor in HAECs an increase in the Bax/Bcl-2 ratio after treatment with L-NAME. This is astonishing, since an increase in the ratio of Bax to Bcl-2 normally indicates an activation of the intrinsic apoptosis pathway [28].

NO is reported to have pro- and antiapoptotic effects depending on its concentration, at which physiological concentrations act antiapoptotic [29, 30]. A study by Liu et al. showed that excessive amounts of NO can lead to an increase in HTRA2/Omi release from mitochondria and endothelial cell apoptosis by formation of peroxynitrite [31]. Here we show that a reduction of physiological NO concentration by inhibition of NO synthesis can result in cytosolic accumulation of HTRA2/Omi. This presumably leads to apoptosis of VSMCs. The locally different eNOS expression we found in BAV patients can account for a lower NO concentration in certain aortic areas and therefore lead to upregulation of HTRA2/Omi which might initiate apoptosis in VSMCs. 

We are aware that our sample number, especially for TAV patients, is limited and our data display a high standard deviation. However, Barker et al. also discovered a high standard deviation when they determined aortic wall shear stress in BAV patients [10]. Hence it could be hypothesized that a high standard deviation in eNOS expression is due to a high standard deviation of aortic wall shear stress which influences expression of eNOS. Fluctuation in shear stress between BAV patients might be due to the morphology of the aortic valve, as BAV is known to feature heterogeneous phenotypes depending on the morphological type of BAV [32–34]. 

In summary, we showed a locally variable eNOS protein expression in BAV aortic aneurysms than in TAV aortic aneurysms. In addition, we showed a cytosolic accumulation of the proapoptotic serine protease HTRA2/Omi after treatment of VSMCs with L-NAME. Taken together, these results indicate a locally different eNOS protein expression, which is probably caused by variations in aortic wall shear stress between BAV and TAV aortic aneurysm patients, which in turn might be caused by differences in BAV and TAV valve morphology. We conclude that eNOS dysregulation can lead to a dysregulation of NO in certain areas of the aorta, which can lead to VSMC apoptosis mediated by HTRA2/Omi. Thus the low eNOS protein expression in the BAV proximal aorta may confer susceptibility to aortic aneurysm formation. 

## Figures and Tables

**Table 1:** Patient characteristics.

Characteristics	BAV (*n* = 14)	TAV (*n* = 5)	*P*-value
Age (years)	51.9 ± 12.2	59.4 ± 8.7	0.257
Sex (f/m)	3/11	1/4	0.5
BMI (kg/m²)	27.5 ± 4.2	28.6 ± 8.2	0.333
Maximum sinus diameter (mm)	38.3 ± 5.1	53.0*	—
STJ diameter (mm)	36.2 ± 4.1	53.0*	—
Tubular diameter (mm)	51.5 ± 4.6	55.4 ± 7.3	0.444
Arch diameter (mm)	39.5 ± 7.3	n.a.	—
Aortic stenosis	6/14 (42.9%)	0/5 (0%)	0.1
Aortic insufficiency	3/14 (21.4%)	5/5 (100%)	0.005
Combined stenosis/insufficiency	5/14 (35.7%)	0/5 (0%)	0.2

BAV: bicuspid aortic valve; BMI: body mass index; f: female; m: male; n.a.: not available; STJ: sinotubular junction; TAV: tricuspid aortic valve; *only data from two patients were available, and therefore statistics were omitted.

**Table 2:** Protein levels of 35 apoptosis-related proteins after treatment of VSMCs with 1 mM L-NAME.

Protein	Control average	1 mM L-NAME average	Change	*P*
Bad	50.59 ± 8.08	54.73 ± 9.86	4.15	0.637
Bax	47.65 ± 14.26	50.98 ± 13.43	3.32	0.805
Bcl-2	31.37 ± 7.47	34.32 ± 5.04	2.95	0.633
Bcl-x	17.62 ± 4.92	19.02 ±6.20	1.40	0.780
Pro-Caspase 3	107.21 ± 20.89	109.96 ± 19.89	2.75	0.889
Cleaved Caspase 3	40.89 ± 12.79	43.35 ± 6.22	2.46	0.799
Catalase	93.40 ± 24.75	97.04 ± 25.26	3.63	0.880
clAP-1	39.80 ± 20.28	45.09 ± 20.87	5.28	0.792
clAP-2	16.72 ± 4.71	18.86 ± 5.27	2.15	0.630
Claspin	40.49 ± 6.13	43.37 ± 6.39	2.89	0.618
Clusterin	22.70 ± 6.30	27.93 ± 7.19	5.23	0.437
Cytochrome c	68.46 ± 8.61	76.39 ± 8.48	7.93	0.336
TRAIL R1/DR4	40.74 ± 6.48	46.36 ± 3.04	5.62	0.241
TRAIL R2/DR5	66.06 ± 16.01	77.34 ± 6.60	11.28	0.363
FADD	64.20 ± 13.56	71.59 ± 4.95	7.39	0.467
Fas/TNFSF6	66.16 ± 23.27	68.26 ± 13.45	2.10	0.904
HIF-1a	29.40 ± 16.62	41.97 ± 15.61	12.57	0.436
HO-1/HMOX1/HSP32	27.06 ± 14.68	35.64 ± 12.58	8.58	0.526
HO-2/HMOX2	38.85 ± 14.23	49.90 ± 18.08	11.04	0.497
HSP27	33.21 ± 7.16	38.64 ± 9.88	5.43	0.528
HSP60	65.84 ± 11.85	70.83 ± 7.05	4.99	0.545
HSP70	66.75 ± 13.76	64.40 ± 5.59	−2.35	0.801
*HTRA2/Omi*	**63.09 ± 3.83**	**72.57 ± 4.18**	**9.48**	0.038*
Livin	14.08 ± 2.52	18.83 ± 5.12	4.75	0.223
PON2	28.81 ± 5.92	31.30 ± 5.53	2.49	0.651
p21/CIP1/CDNK1A	27.93 ± 9.01	38.74 ± 9.16	10.81	0.259
p27/Kip1	16.75 ± 6.04	21.34 ± 2.89	4.59	0.345
Phospho-p53 (S15)	82.25 ± 19.34	88.18 ± 11.03	5.93	0.696
Phospho-p53 (S46)	64.83 ± 18.94	71.42 ± 20.27	6.59	0.729
Phospho-p53 (S392)	64.02 ± 28.92	75.33 ± 22.81	11.31	0.659
Phospho-Rad17 (S635)	11.71 ± 2.21	18.72 ± 5.65	7.01	0.129
SMAC/Diablo	56.47 ± 11.12	59.84 ± 9.98	3.36	0.742
Survivin	57.52 ± 23.63	61.96 ± 19.33	4.44	0.831
TNF RI/TNFRSF1A	18.18 ± 4.27	22.05 ± 1.64	3.87	0.210
XIAP	49.96 ± 5.39	56.28 ± 3.45	6.32	0.149

All values are in percent of the calibrator (positive control). The change is given in percent points of the calibrator. The data were acquired in three independent experiments. *: *P* < 0.05.

**Table 3:** Protein levels of 35 apoptosis-related proteins after treatment of HAECss with 1 mM L-NAME.

Protein	Control average	1 mM L-NAME average	Change	*P*
Bad	62.83 ± 4.88	66.76 ± 12.67	3.92	0.661
Bax	52.36 ± 16.36	54.62 ± 12.14	2.26	0.878
Bcl-2	35.77 ± 6.52	40.28 ± 4.95	4.51	0.373
Bcl-x	20.18 ± 5.18	17.03 ± 3.48	−3.16	0.410
Pro-Caspase 3	92.79 ± 10.67	89.73 ± 9.55	−3.06	0.756
Cleaved Caspase 3	29.29 ± 7.84	23.81 ± 3.32	−5.48	0.392
Catalase	58.39 ± 12.35	55.87 ± 6.19	−2.53	0.803
clAP-1	35.59 ± 9.16	27.73 ± 16.97	−7.87	0.560
clAP-2	15.76 ± 3.62	16.86 ± 5.57	1.10	0.813
Claspin	18.69 ± 2.87	18.00 ± 2.51	−0.69	0.745
Clusterin	35.62 ± 11.69	39.72 ± 4.57	4.10	0.660
Cytochrome c	62.37 ± 11.07	84.29 ± 6.92	21.92	0.069
TRAIL R1/DR4	56.01 ± 3.15	57.91 ± 12.80	1.91	0.833
TRAIL R2/DR5	88.48 ± 1.03	91.83 ± 8.18	3.35	0.516
FADD	72.14 ± 8.15	72.21 ± 7.40	0.07	0.989
Fas/TNFSF6	41.58 ± 7.37	34.54 ± 9.89	−7.04	0.303
HIF-1a	28.35 ± 7.46	26.07 ± 6.38	−2.29	0.735
HO-1/HMOX1/HSP32	30.43 ± 6.38	26.90 ± 12.93	−3.53	0.728
HO-2/HMOX2	35.86 ± 1.94	34.72 ± 11.64	−1.15	0.887
HSP27	41.67 ± 9.25	44.93 ± 11.15	3.26	0.699
HSP60	62.68 ± 8.65	70.54 ± 22.29	7.87	0.640
HSP70	65.35 ± 7.42	69.88 ± 17.35	4.53	0.727
HTRA2/Omi	78.98 ± 5.64	84.13 ± 9.00	5.15	0.482
Livin	12.39 ± 2.85	17.58 ± 6.45	5.18	0.315
PON2	41.23 ± 2.47	44.50 ± 12.63	3.27	0.711
p21/CIP1/CDNK1A	30.05 ± 11.24	35.52 ± 9.31	5.47	0.611
p27/Kip1	30.42 ± 3.67	29.70 ± 4.48	−0.73	0.777
Phospho-p53 (S15)	22.92 ± 6.58	25.48 ± 6.03	2.56	0.680
Phospho-p53 (S46)	8.84 ± 4.14	9.08 ± 2.81	0.24	0.948
Phospho-p53 (S392)	4.06 ± 2.69	5.01 ± 6.63	0.95	0.844
Phospho-Rad17 (S635)	12.24 ± 3.83	13.16 ± 9.86	0.92	0.900
SMAC/Diablo	63.31 ± 14.25	63.90 ± 12.04	0.59	0.956
Survivin	23.28 ± 1.48	21.28 ± 3.96	−2.00	0.443
TNF RI/TNFRSF1A	27.19 ± 3.24	25.24 ± 4.74	−1.95	0.599
XIAP	53.37 ± 7.73	51.67 ± 3.93	−1.70	0.769

All values are in percent of the calibrator (positive control). The change is given in percent points of the calibrator. The data were acquired in three independent experiments.
